# Identification of a Conserved B-cell Epitope on Reticuloendotheliosis Virus Envelope Protein by Screening a Phage-displayed Random Peptide Library

**DOI:** 10.1371/journal.pone.0049842

**Published:** 2012-11-21

**Authors:** Mei Xue, Xingming Shi, Jing Zhang, Yan Zhao, Hongyu Cui, Shunlei Hu, Hongbo Gao, Xianlan Cui, Yun-Feng Wang

**Affiliations:** 1 Division of Avian Infectious Diseases, State Key Laboratory of Veterinary Biotechnology, Harbin Veterinary Research Institute, The Chinese Academy of Agricultural Sciences, Harbin, P. R. China; 2 Animal Health Laboratory, Department of Primary Industries, Parks, Water and Environment, Tasmania, Australia; University of Alabama at Birmingham, United States of America

## Abstract

**Background:**

The gp90 protein of avian reticuloendotheliosis-associated virus (REV-A) is an important envelope glycoprotein, which is responsible for inducing protective antibody immune responses in animals. B-cell epitopes on the gp90 protein of REV have not been well studied and reported.

**Methods and Results:**

This study describes the identification of a linear B-cell epitope on the gp90 protein by screening a phage-displayed 12-mer random peptide library with the neutralizing monoclonal antibody (mAb) A9E8 directed against the gp90. The mAb A9E8 recognized phages displaying peptides with the consensus motif SVQYHPL. Amino acid sequence of the motif exactly matched ^213^SVQYHPL^219^ of the gp90. Further identification of the displayed B cell epitope was conducted using a set of truncated peptides expressed as GST fusion proteins and the Western blot results indicated that ^213^SVQYHPL^219^ was the minimal determinant of the linear B cell epitope recognized by the mAb A9E8. Moreover, an eight amino acid peptide SVQYHPLA was proven to be the minimal unit of the epitope with the maximal binding activity to mAb A9E8. The REV-A-positive chicken serum reacted with the minimal linear epitopes in Western blot, revealing the importance of the eight amino acids of the epitope in antibody-epitope binding activity. Furthermore, we found that the epitope is a common motif shared among REV-A and other members of REV group.

**Conclusions and Significance:**

We identified ^213^SVQYHPL^219^ as a gp90-specific linear B-cell epitope recognized by the neutralizing mAb A9E8. The results in this study may have potential applications in development of diagnostic techniques and epitope-based marker vaccines against REV-A and other viruses of the REV group.

## Introduction

Reticuloendotheliosis viruses (REVs) are a group of viruses in the family *Retroviridae*, specifically gammaretroviruses in the same genus as mammalian C-type retroviruses [1]. The REV group includes defective REV-T [2], [3], non-defective REV-A [4], [5], chick syncytial virus (CSV) [6], duck infectious anemia virus [7], and spleen necrosis virus (SNV) [8]. Except for the defective REV-T, all isolated REV strains belong to a single serotype [5] and their genetic sequences show little variation [9].

REV genome consists of three structural genes (*gag*, *pol* and *env*) flanked by long-terminal repeats (LTRs) [10]. The major mature *env* gene products of REVs are the surface glycoprotein (gp90) and the transmembrane protein (gp20) [11], [12]. The gp90 protein containing both continuous and discontinuous epitopes functions as the immunodominant protein [13] and is responsible for eliciting REV antibodies. Previous studies indicated that the C-terminal epitope of gp90 was exposed on the outer surface of the REV-A-infected cell [12]. However, the epitope identified in REV gp90 protein has not been finely mapped, and the core sequence of the epitope needs to be determined.

Detailed analysis of epitopes is important for the understanding of immunological events, and the development of epitope-based marker vaccines and diagnostic tools for various diseases [14], [15]. In this study, we prepared a neutralizing monoclonal antibody (mAb) against gp90 protein from the REV-A strain HLJ07I, and used it to screen a phage-displayed random 12-mer peptide library for the linear B-cell epitope. This study describes the first identification of the precise location of the epitope on gp90 protein. The information provided in this study will facilitate the development of specific serological diagnosis of REV infection, and will contribute to the rational design of vaccines by further understanding of the antigenic structure of gp90.

## Materials and Methods

### Ethics Statement

Care of laboratory animals and animal experimentation were performed in accordance with animal ethics guidelines and approved protocols. All animal studies were approved by the Animal Ethics Committee of Harbin Veterinary Research Institute of the Chinese Academy of Agricultural Sciences (SYXK (H) 2006-032).

### Viruses and Cells

REV-A Strain HLJ07I (GenBank accession No. GQ375848) was isolated from Heilongjiang Province in China in 2007. Chicken embryo fibroblasts (CEFs) were prepared as primary cultures from 10-day-old chicken embryos as previously described [16] and were grown in Dulbecco's modified Eagle's medium (DMEM) supplemented with 10% fetal bovine serum plus antibiotics. Viruses were grown in CEFs and incubated at 37°C with 5% CO_2_ for 5 days. The suspension was frozen and thawed three times to disrupt cells and release virus, and then clarified by two centrifugation steps (2000 g for 15 min, and 10,000 g for 60 min). Virus present in the upper phase was precipitated with 10% (w/v) polyethylene glycol 6000 (PEG 6000) for 4 hours at 4°C. Precipitates were collected by centrifugation at 9,000 g for 30 minutes and resuspended in TNE buffer (50 mM tris-HC1, pH 7.5; 0.1 M NaC1, 10 mM EDTA). Finally, they were centrifuged through a 30% (w/v) sucrose cushion for 90 minutes at 200,000 g and resuspended in TNE buffer. The purified virus was analyzed in SDS-PAGE.

### MAb Production and Characterization

Six-week-old female BALB/c mice were subcutaneously immunized with 100 µg of the purified recombinant gp90 protein emulsified with an equal volume of Freund’s complete adjuvant (Sigma, St. Louis, MO, USA). Two boosters of the Freund’s incomplete adjuvant (Sigma, St. Louis, MO, USA) emulsified antigen were given at two week interval. Two weeks after the third immunization, the mice were intraperitoneally boosted with 100 µg antigen alone. Three days later, the spleen cells from immunized mice were fused with myeloma cells SP2/0 (SP2/0-Agl4; ATCC CRL 1581) [17], using 50% (wt/vol) polyethylene glycol and 10% dimethyl sulfoxide (DMSO) (vol/vol) (Sigma, St Louis, MO, USA). Hybridomas were screened by indirect enzyme-linked immunosorbent assay (ELISA) and indirect immunofluorescence assay (IFA). The hybridomas producing mAbs were cloned three times by limiting dilution of the cells. Antibody subtype identification was performed using SBA Clonotyping™ System/HRP Kit (Southern Biotech, Birmingham, AL, USA).

### Indirect ELISA

Plates were coated with 100 µL/well of purified REV gp90 antigen diluted in carbonate-bicarbonate buffer (pH 9.6) for incubation overnight at 4°C. Following 4 washes with 200 µL/well of PBS/0.05% Tween-20, the plates were blocked with 200 µL/well of blocking buffer (PBS containing 5% skim milk) for 1 h at 37°C. The supernatant of hybridoma culture (100 µL/well) was added in duplicate and the plates were incubated for 1 h at 37°C. After washing three times with PBS, 100 µL of horseradish peroxidase (HRP)-conjugated goat anti-mouse immunoglobulin G (IgG, 1∶5,000 dilution,Sigma, St Louis, MO, USA) was added to each well and incubated for 1 h at 37°C. Plates were washed three times with PBS and incubated with 100 µL/well of o-phenylenediamine dihydrochloride (OPD, Sigma, St Louis, MO, USA) containing 0.3% H_2_O_2_ for 5 minutes at room temperature in the dark. The reaction was stopped with 50 µL/well of 2 M H_2_SO_4_ and the absorbance measured at 492 nm.

### Indirect Immunofluorescence Assay

About 70–80% confluent CEF cells in 96-well plates were infected with REV-A HLJ07I at a MOI of 0.2. At 5 days post-infection, the infected cells were fixed with icy cold ethanol absolute for 15 min at 4°C, and air dried. The fixed cells were incubated with mAb A9E8, REV-A-positive chicken serum, anti-porcine IFN-γ mAb (Sigma, St Louis, MO, USA), or REV-A-negative chicken serum for 1 h at 37°C. After washing three times with PBS, 50 µL/well of FITC-conjugated goat anti-mouse IgG or FITC-conjugated rabbit anti-chicken IgG (Sigma, St Louis, MO, USA) at 1∶100 dilutions were added and incubated for 1 h at 37°C. The cells were rinsed three times with PBS and once with deionized water, and mounted in 50 µL of 90% glycerol in PBS, and then observed under the Nikon Eclipse Ti-E microscope equipped with NIS-Elements AR software.

### Micro-neutralization Assay

The micro-neutralization assay was modified from a previously described procedure [18]. The ascitic fluid was heat inactivated for 30 min at 56°C, and two fold serial dilutions were incubated with 2×10^3^ tissue culture infective doses 50% (TCID_50_/mL ) of REV-A in a 96-well micro-plate. Four uninfected control wells were included on each plate as control wells. After 2 h incubation at 4°C, 100 µL of CEF cells at 1.5×10^5^ cells/mL was added to each well. The plates were incubated for 5 days at 37°C and 5% CO_2_. The monolayers were washed with PBS and fixed in icy cold ethanol for 15 minutes. The presence of viral gp90 protein was detected by ELISA with the mAb A9E8. The absorbance was measured at 492 nm with an ELISA microplate reader. The average A492 was determined for quadruplicate wells of virus-infected and uninfected control wells, and a neutralizing endpoint was determined by using a 50% specific signal calculation. The endpoint titer was expressed as the reciprocal of the highest dilution of ascitic fluid with A492 value less than X, where × = [(average A492 of infected wells) − (average A492 of control wells)]/2+ (average A492 of control wells).

### Biopanning

The Ph.D.-12™ Phage Display Peptide Library Kit was purchased from New England BioLabs Inc. The dodecapeptide library consisted of 2.7×10^9^ electroporated sequences (1.5×10^13^ pfu/mL). The mAb was purified from the ascites ﬂuid of mice inoculated with the hybridma cells secreting A9E8 by affinity chromatography using rProtein G Agorose (Invitrogen, Carlsbad, CA,USA) according to the manufacturer’s instructions. The concentration of the purified protein was determined using the Bradford Protein Assay Kit (Beyotime, Shanghai, China). Three successive rounds of biopanning were carried out according to the manufacturer’s instruction manual. Brieﬂy, one well of a 96-well microtiter plate was coated with 10 µg/mL of mAb A9E8 in coating buffer (0.1 M NaHCO_3_, pH 8.6) overnight at 4°C, followed by blocking with blocking buffer (0.1 M NaHCO_3_, pH 8.6, 0.02% NaN3, and 5 mg/ml BSA) for 2 h at 4°C. The phage library (1.5×10^11^ phages/100 µL) was added to the blocked wells and the plate incubated for 1 h at room temperature. The unbound phages were removed by successive washings with TBS buffer (50 mM Tris–HCl, pH 7.5, 150 mM NaCl) containing gradually increased concentrations (0.1%, 0.3%, and 0.5%) of Tween-20, and the bound phages were eluted by 0.2 M glycine-HCl containing 1 mg/mL BSA (pH 2.2) and immediately neutralized with 1 M Tris-HCl (pH 9.1). The eluted phages were amplified by infecting *E. coli* (ER2738), and were titered on LB/IPTG/Xgal plates for the subsequent rounds of selection. The output to input ratio was calculated as follows: (titer of the amplified eluent phages/titer of the input phages (1.5×10^11^))×100%.

### Phage ELISA

After three rounds of biopanning, eight individual phage clones were selected for target binding in ELISA as described in the manufacturer’s instructions. Brieﬂy, 96-well plates were coated with 100 ng of purified mAb A9E8, or anti-porcine IFN-γ mAb (Sigma, St Louis, MO, USA) as negative controls overnight at 4°C. The coated wells were blocked for 2 h at room temperature and then the phages (10^10^ pfu/100 µL/well) diluted in blocking solution were added. The plates were incubated for 1 h at room temperature followed by washing ten times with TBST. Bound phages were subjected to reaction with horseradish peroxidase (HRP)-conjugated sheep anti-M13 antibody (Pharmacia, Piscataway, NY, USA), followed by color development with substrate solution containing o-phenylenediamine (OPD).

### Sequencing of DNA Inserts Displayed by Phage Clones

The positive phage clones identified by phage ELISA were sequenced with the −96 gIII sequencing primer 5′-TGA GCG GAT AAC AAT TTC AC-3′ as described in the manufacturer’s instructions.

### Construction of Expression Plasmids and GST Fusion Expression in *E. coli*

A series of complementary oligonucleotides (Table 1) coding for wild-type and truncated motif SVQYHPL were synthesized, annealed, and cloned into the BamHI/XhoI sites of the prokaryotic expression vector pGEX-6p-1 (Pharmacia, Piscataway, NY, USA), producing a group of recombinant plasmids. All the resulting recombinant plasmids were validated by restriction analysis and DNA sequencing. Expression plasmids were transformed into BL21 (DE3) competent cells, followed by the addition of 1 mM isopropyl-D-thioga-lactopyranoside (IPTG; GE Healthcare, USA) for induction.

**Table 1:** The oligonucleotides coding for the wild-type and truncated versions of the epitope SVQYHPL.

Name	The sequences of oligonucleotides	Coding motifs (designations)
H7wt-S	*GATCC* TCCGTACAGTATCACCCTTTATAA*C*	SVQYHPL(H7wt)
H7wt-R	*TCGAG* TTATAAAGGGTGATACTGTACGGA*G*	
H7ΔS-S	*GATCC* GTACAGTATCACCCTTTATAA*C*	-VQYHPL(H7ΔS)
H7ΔS-R	*TCGAG* TTATAAAGGGTGATACTGTAC*G*	
H7ΔL-S	*GATCC* TCCGTACAGTATCACCCTTAA*C*	SVQYHP-(H7ΔL)
H7ΔL-R	*TCGAG* TTAAGGGTGATACTGTACGGA*G*	
H7ΔSV-S	*GATCC* CAGTATCACCCTTTATAA*C*	–QYHPL(H7ΔSV)
H7ΔSV-R	*TCGAG* TTATAAAGGGTGATACTG*G*	
H7ΔPL-S	*GATCC* TCCGTACAGTATCACTAA*C*	SVQYH-(H7ΔPL)
H7ΔPL-R	*TCGAG* TTAGTGATACTGTACGGA*G*	
R1-S	*GATCC* TCCGTACAGTATCACCCTTTAGCCTAA*C*	SVQYHPLA(R1)
R1-R	*TCGAG* TTAGGCTAAAGGGTGATACTGTACGGA*G*	
R2-S	*GATCC* TCCGTACAGTATCACCCTTTAGCCCTGTAA*C*	SVQYHPLAL(R2)
R2-R	*TCGAG* TTACAGGGCTAAAGGGTGATACTGTACGGA*G*	
R3-S	*GATCC* TCCGTACAGTATCACCCTTTAGCCCTGCCCTAA*C*	SVQYHPLALP(R3)
R3-R	*TCGAG* TTAGGGCAGGGCTAAAGGGTGATACTGTACGGA*G*	
L1-S	*GATCCCCC* TCCGTACAGTATCACCCTTTATAA*C*	PSVQYHPL(L1)
L1-R	*TCGAG* TTATAAAGGGTGATACTGTACGGAGGG*G*	
L2-S	*GATCCTACCCC* TCCGTACAGTATCACCCTTTATAA*C*	YPSVQYHPL(L2)
L2-R	*TCGAG* TTATAAAGGGTGATACTGTACGGAGGGGTA*G*	
L3-S	*GATCCAGCTACCCC* TCCGTACAGTATCACCCTTTATAA*C*	SYPSVQYHPL(L3)
L3-R	*TCGAG* TTATAAAGGGTGATACTGTACGGAGGGGTAGCT*G*	

Notes: Introduced bases for cloning (to form the overhanging ends of BamHI and XhoI after annealing the two complementary oligonucleotides) are shown in italics; stop codons are boxed; deleted residues are shown as dashes; and the designations of the motifs are shown in parentheses.

### SDS-PAGE and Western Blot

Approximately equivalent amount of each GST fusion protein was subjected to 12% sodium dodecyl sulfate-polyacrylamide gel electrophoresis (12% SDS–PAGE). The gel was either stained with commassie blue staining solution or electrophoretically transferred to nitrocellulose membrane. After being blocked with 5% skim milk in PBS overnight at 4°C, the membrane was incubated with mAb A9E8 (diluted 1∶2,000 in PBS) or REV-A-positive chicken serum (diluted 1∶100 in PBS) at 37°C for 1 h. After being washed three times with PBST, the membrane was probed with a 1∶5,000 dilution of HRP-conjugated goat anti-mouse IgG (Sigma, St Louis, MO, USA) or HRP-conjugated rabbit anti-chicken IgG (Sigma, St Louis, MO, USA) at 37°C for 1 h. Reactivity was visualized with the substrate 3, 3'-diaminobenzidine (DAB; Sigma, St Louis, MO, USA).

### Homology Analysis

To investigate the conservation of the epitope among REV viruses, sequence alignment of the epitope and the corresponding regions on gp90 proteins of 32 REV-A strains, one REV-T strain, four SNV strains and one CSV strain was performed using the DNASTAR Lasergene program (Windows version; DNASTAR Inc., Madison, WI, USA).

## Results

### Generation and the Neutralization Titer of Neutralizing mAb

**Figure 1:**
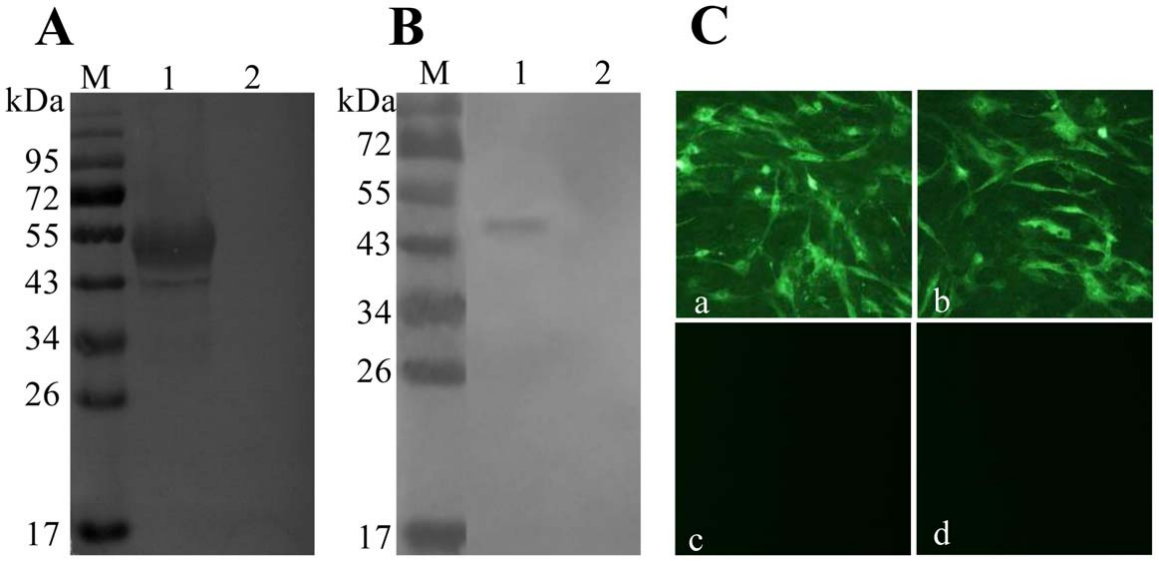
The binding of mAb A9E8 with recombinant gp90 protein, the purified whole virus and REV-A infected CEF cells. (A) Reactivity of the mAb A9E8 with recombinant gp90 protein. Lane 1, cell lysates of *E. coli* BL21 (DE3) harboring pET-REV-A-gp90; Lane 2, cell lysates of *E. coli* BL21 (DE3) harboring pET-32a. (B) Reactivity of the mAb A9E8 with the purified whole virus. Lane 1, purified virus; Lane 2, supernatant from CEF cells. (C) Analysis of ethanol-fixed REV-A infected CEF cells by immunofluourescence with (a) mAb A9E8, (b) a positive control serum (REV-A-positive chicken serum), (c) a negative control mAb (anti-porcine IFN-γ mAb), and (d) a negative control serum (REV-A-negative chicken serum).

Purified gp90 protein was used to immunize BALB/c mice. After cell fusion and screening, several hybridoma cell lines were generated, which produced gp90-reactive mAbs. One monoclonal antibody produced by the line designated as A9E8 was selected for strong reactivity with recombinant gp90 protein in Western blot (Figure 1A) and in an indirect ELISA (data not shown). It also showed strong reactivity with purified whole virus in Western blot (Figure 1B) and could be used to detect REV-A antigen by an indirect immunofluorescence assay (IFA; Figure 1C). The mAb A9E8 was compose of an IgG2b heavy chain paired with a κ-type light chain, as determined using the SBA Clonotyping™ System/HRP Kit. The titers of antibody in hybridoma cell culture supernatants and in ascites were measured by indirect ELISA and determined to be 1∶3,200 and 1∶128,000, respectively.

The neutralizing activities of the mAb A9E8 were then determined by a micro-neutralization assay on CEF cells using REV-A HLJ07I. The mAb A9E8 neutralized the virus with a neutralization titer (NT_50_) of 100.

### Phage Enrichment by Biopanning

To determine the epitope recognized by mAb A9E8, biopanning of a phage displayed 12-mer random peptide library was performed using the affinity purified mAb A9E8. After three rounds of biopanning, an enrichment of phages bound to the mAb A9E8 was obtained. The output to input ratios of the three rounds of biopanning were 0.00008%,0.038% and 0.79%.

### Epitope Prediction

**Figure 2:**
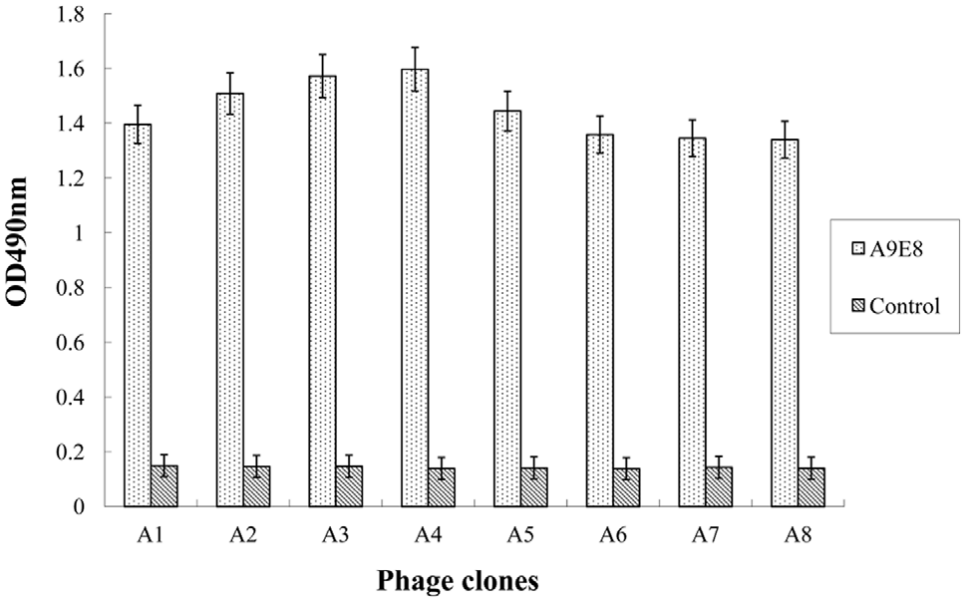
Detection of the selected phages for antibody binding by Phage ELISA. Eight phage clones selected after three rounds of biopanning were added to the microplate wells (10^10^ pfu/100 µL/well) coated with the mAb A9E8 or anti-porcine IFN-γ mAb (negative control) (100 ng/well ), and incubated for 1 h at room temperature. Bound phages were subjected to reaction with horseradish peroxidase (HRP)-conjugated anti-M13 antibody, followed by color development with substrate solution containing o-phenylenediamine (OPD). Three independent assays were performed for each selected phage.

Eight phage clones were selected for reactivity with the mAb A9E8 after three rounds of biopanning and enrichment of the phages binding to the mAb A9E8. These selected clones were further evaluated by Phage ELISA for reactivity with the mAb A9E8 and a negative control mAb (anti-porcine IFN-γ). As shown in Figure 2, all the selected eight phage clones (A1–A8) showed specific reactivity with A9E8 (OD492 nm >1.10), but not with anti-porcine IFN-γ mAb (OD492 nm <0.15). The eight phage clones were sequenced, and were shown to display a consensus sequence SVQYHPL, which was identical to the motif ^213^SVQYHPL^219^ at the C-terminus of the gp90 protein of REV-A strain HLJ07I (Table 2).

**Table 2:** Sequence comparison of random peptide inserts displayed on the positive phages.

Phage	Amino acid sequence of the insert^a^																		
A1						**S**	Y	**Q**	**Y**	**H**	T	**L**	T	Y	T	E	L		
A2			G	M	A	G	**V**	**Q**	**Y**	**H**	**P**	**L**	H	L					
A3	V	N	A	L	Y	**S**	**V**	**Q**	**Y**	Q	**P**	**L**							
A4			S	Y	L	**S**	**V**	**Q**	**Y**	E	**P**	**L**	L	T					
A5					A	**S**	**V**	D	**Y**	Y	T	**L**	T	D	L	R			
A6	L	E	K	F	N	M	**V**	**Q**	G	Q	H	T							
A7					A	Y	**V**	T	**Y**	T	**P**	**L**	Y	T	T	A			
A8						**S**	L	**Q**	**Y**	S	Y	Y	Y	E	E	Y	Y		
Consensus						**S**	**V**	**Q**	**Y**	**H**	**P**	**L**							
REV gp90	R	H	S	Y	P	**S**	**V**	**Q**	**Y**	**H**	**P**	**L**	A	L	P				

a Conservative amino acid motifs are bold and underlined.

### Precise Defining of the Epitope

**Figure 3:**
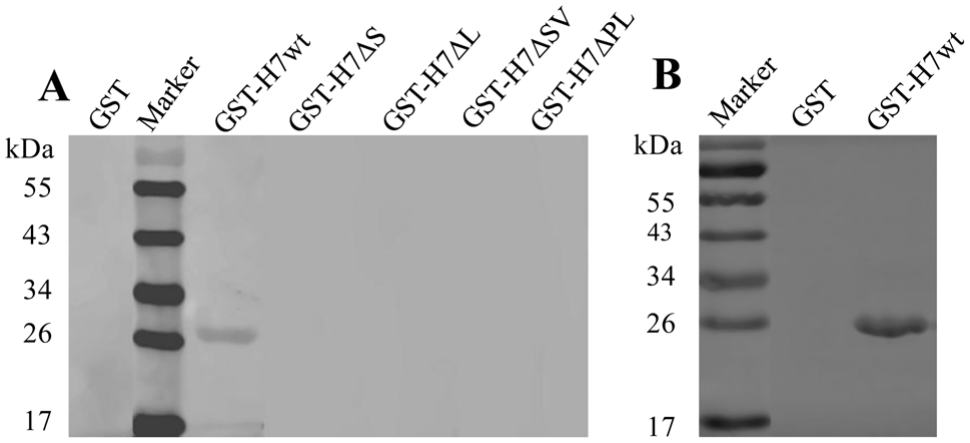
Reactivity of the GST-fusion proteins containing truncated motifs derived from the epitope ^213^SVQYHPL^219^ with the mAb A9E8 or REV-A-positive chicken serum in Western blot. (A) Reactivity of fusion proteins with mAb A9E8. Each GST fusion was electrophoresed on 12% SDS-PAGE. The proteins were transferred onto nitrocellulose membranes. The membranes were incubated with the mAb A9E8. Reactions were detected using HRP-conjugated goat anti-mouse IgG, and the color was developed using 3,3-diaminobenzidine tetrahydrochloride (DAB) and stopped by rinsing in deionized water. Marker: PageRuler™ Prestained Protein Ladder. GST-H7wt: fusion containing the motif SVQYHPL; GST-H7ΔS: VQYHPL; GST-H7ΔL: SVQYHP; GST-H7ΔSV: QYHPL; GST-H7ΔPL: SVQYH. (B) Reactivity of antibodies in REV-A-positive chicken serum with the GST fusion protein containing the SVQYHPL epitope. GST alone or GST fused with the SVQYHPL peptide epitope (GST-H7wt) was evaluated by Western blot analysis for reactivity with antibody in REV-A-positive chicken serum.

To verify whether the identified motif represented an epitope recognized by the mAb A9E8, a DNA fragment coding for the motif SVQYHPL was expressed as a GST fusion protein (GST-H7wt) in *E. coli*. Western blot analysis showed that the fusion protein was recognized by the mAb A9E8 (Figure 3A) and REV-A infected chicken antiserum (Figure 3B), indicating that the motif represented a linear B-cell epitope.

To define the epitope precisely, four mutants with deletions at C- and N-termini of the motif SVQYHPL (Table 1) were constructed to express the GST fusions GST-H7ΔS, GST-H7ΔL, GST-H7ΔSV, and GST-H7ΔPL representing -VQYHPL, SVQYHP-, –QYHPL and SVQYH– (deletions were shown as dashes) in *E. coli*, respectively. We found that only the full-length SVQYHPL polypeptide (GST-H7wt) was recognized by the mAb A9E8 (Figure 3A). Removal of one or more amino acids at either the amino or carboxyl terminus of the peptide abolished antibody binding, indicating that the peptide SVQYHPL represented the minimal requirement for the reactivity of the epitope with A9E8.

### Minimal Unit of the Epitope with the Maximal Binding Activity to mAb A9E8

**Figure 4:**
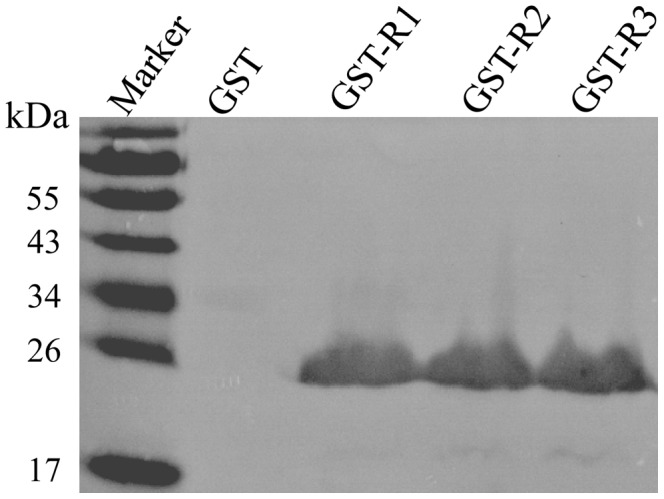
Determination of the minimal epitope unit with the maximal binding activity to mAb A9E8 by Western blot. Marker: PageRuler™ Prestained Protein Ladder. GST-R1: fusion containing the motif SVQYHPLA; GST-R2: SVQYHPLAL; GST-R3: SVQYHPLALP.

To investigate minimal unit of the epitope with the maximal binding activity to mAb A9E8, a series of GST-fusion proteins were expressed with extended amino acid residues at both N and C termini of the motif SVQYHPL (Table 1). These GST-fusion proteins were subjected to SDS-PAGE and testing for reactivity with mAb A9E8 in Western blot. Fusion proteins GST-R1 (SVQYHPLA), GST-R2 (SVQYHPLAL) and GST-R3 (SVQYHPLALP) reacted strongly with mAb A9E8 in Western blot (Figure 4). The GST-R2 and GST-R3 showed similar binding activity to the GST-R1, indicating that alanine alone significantly increased binding activity of the core epitope to mAb A9E8. In contrast, GST-fusion proteins with extended amino acid residues at the N terminus of the motif SVQYHPL showed no increased binding activity compared with GST-H7wt in Western blot (data not shown). Taken together, these results showed that SVQYHPLA was the minimal unit of the epitope with the maximal binding activity to mAb A9E8.

### SVQYHPL is a Highly Conserved Epitope among All REV Strains

**Figure 5:**
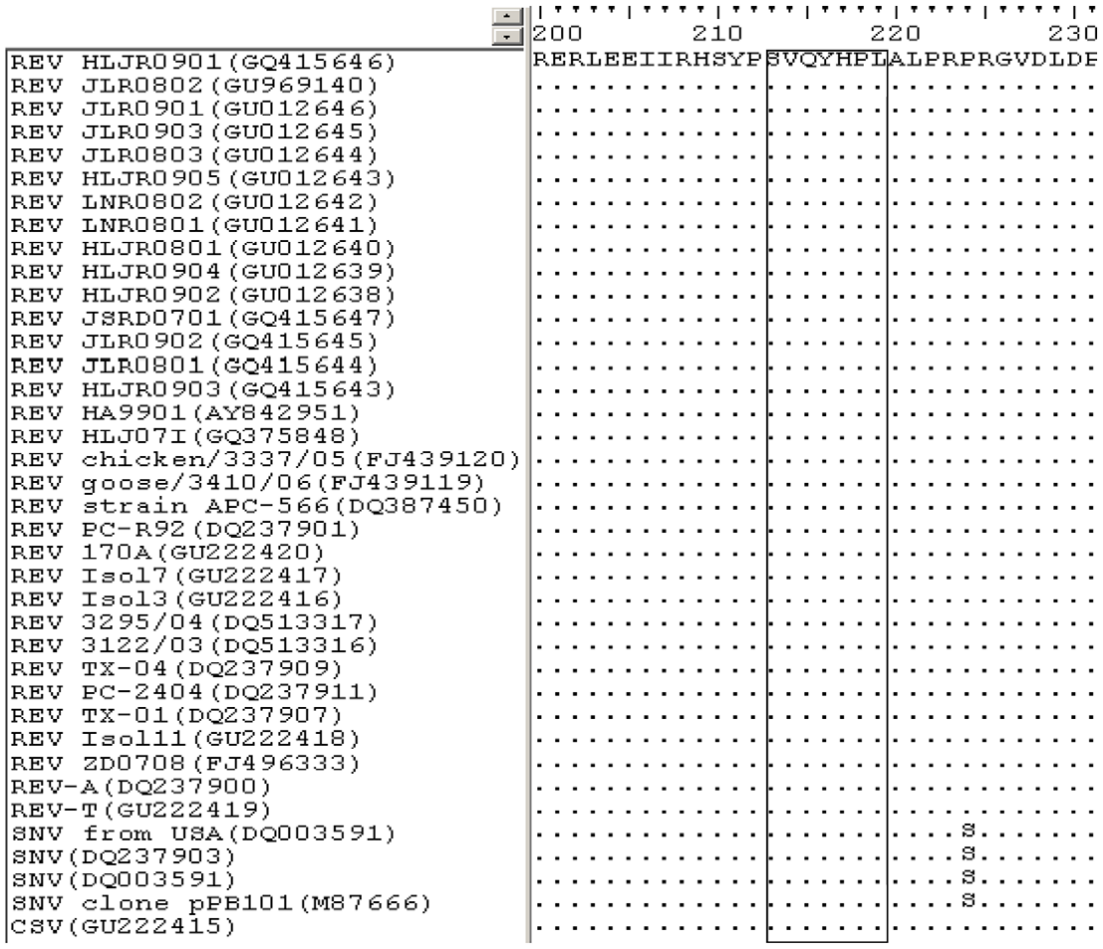
Alignment of the sequences of 32 REV-A strains, one REV -T strain and 5 other reticuloendotheliosis virus strains surrounding the epitope-coding region on the gp90 protein. The GenBank accession numbers of the REV strains used are indicated in parentheses. The homologous sequences of different REVs strains corresponding to the identified epitope are boxed. Dashes indicate identical bases.

To investigate the conservation of the SVQYHPL epitope, we aligned the epitope identified in this study with REVs gp90 coding regions available in GenBank. The alignment results showed that all amino acids in the motif were identical among all REV strains (Figure 5), indicating that the motif represented a conserved epitope on the gp90 protein of REVs.

## Discussion

The gp90 protein of REV is an important antigenic protein and is associated with virus neutralization, which is the major candidate antigen for vaccine development and disease serological diagnosis [12], [13]. Studies showed that recombinant gp90 protein expressed in *Pichia pastoris* induced a protective immune response against REV in chickens [19]. Precise mapping of epitopes in gp90 is important for understanding antibody-mediated protection and developing epitope-based marker vaccines and diagnostic tools. Cui *et al.* [20] reported the generation and partial characterization of a panel of 11 mAbs against the nondefective REV Strain T, and showed that the epitope was on the viral envelope glycoprotein. However, they only identified the relative regions in REV envelope glycoprotein recognized by the mAbs, and did not map the fine locations of the epitopes. To our knowledge, there has been no report on linear epitope mapping of the gp90 of REV.

Mapping epitopes using monoclonal antibodies has become a powerful tool to study protein structure and has been used to diagnose diseases and design marker vaccines [21], [22], [23]. In this study, we described the generation and epitope mapping of a gp90 protein specific mAb, and demonstrated that the epitope was conserved among the REV group. Precise analysis of REV-A gp90 protein epitope will provide the fundamental information for development of epitope-based vaccines and diagnostic tools for REV-A and/or other REV group infection.

Phage display is an *in vitro* selection technique in which a peptide or protein is genetically fused to a coat protein of bacteriophage and the fused peptide or protein is displayed on the exterior surface of the phage virion. The phage displayed random peptide library is a powerful and high throughput tool for rapid mapping of epitopes [24].

In this study, we generated a gp90-specific mAb A9E8 using recombinant gp90 protein expressed in *E. coli*. The mAb A9E8 showed strong reactivity against purified whole virus in Western blot and could be used to detect REV-A antigen by an indirect immunofluorescence assay. The linear epitope recognized by the mAb A9E8 was defined as SVQYHPL by screening a random phage display peptide library. This peptide sequence was identical to ^213^SVQYHPL^219^ of the gp90 protein of REV-A. N- or C-terminal deletions of amino acids of this epitope demonstrated that ^213^SVQYHPL^219^ is the minimal requirement for recognition by A9E8. Fusion proteins GST-R1 with extended amino acid residues at the C terminus of the motif SVQYHPL showed increased binding activity compared with that of GST-H7wt in Western blot, indicating that alanine alone significantly increased binding activity of the core epitope to mAb A9E8. Thus, the peptide SVQYHPLA was determined to be the minimal unit of the epitope with the maximal binding activity to mAb A9E8.

The peptide was also recognized by REV-A-positive chicken serum, revealing the importance of the eight amino acids of the epitope in antibody-epitope binding reactivity. Sequence alignments of REV-A strains, REV-T strain and five other REV strains demonstrated that the motif was highly conserved among REV viruses, indicating that it is a broad group-specific epitope. Since A9E8 was identified as a neutralizing mAb, the epitope identified with A9E8 in this study was a neutralizing epitope.

Many neutralizing epitopes have been mapped in the variable regions of the proteins of viruses, including infectious bursal disease virus [25], infectious bronchitis virus [26], hepatitis C virus [27], and HIV [28]. Some neutralizing epitopes, however, are highly conserved across most of the viruses in the same group [29], [30]. A novel epitope was mapped within the highly conserved flavivirus fusion loop peptide ^98^DRXW^101^ by phage-display biopanning and structure modeling using mAb 2A10G6 that had broad cross-reactivity with dengue virus (DENV) 1–4, yellow fever virus (YFV), West Nile virus (WNV), and Japanese encephalitis virus (JEV) viruses. This mAb potently neutralized DENV 1–4, YFV, and WNV and conferred protection against lethal challenge with DENV 1–4 and WNV in murine model. Further functional studies revealed that 2A10G6 blocked infection at a step after viral attachment. These results show that the broad cross-reactivity epitope recognized by neutralizing mAb 2A10G6 is highly conserved among DENV 1–4, YFV and WNV [29]. An epitope recognized by mAb 51 belonging to isotype IgM was mapped to ^215^KQEKD^219^ of the VP1 capsid protein of Enterovirus 71 (EV71), which possessed neutralizing activity *in vitro* and provided 100% *in vivo* passive protection against lethal challenge with EV71 strain HFM 41. BLAST analyses of the neutralizing epitope revealed that it was highly conserved among all EV71 strains, but not coxsachievirus 16 [30]. In this study, the epitope recognized by neutralizing mAb A9E8 was mapped to a highly conserved region of the gp90 protein among REVs, which would be useful for development of REV marker vaccines and diagnostic techniques.

### Conclusions

In summary, a highly conserved neutralizing linear B-cell epitope on the gp90 protein of REV-A was identified in this study. The identified conserved epitope may have potential for development of REV specific diagnostic assays and epitope-based marker vaccines.
